# Experts’ views on the implementation of digital interventions for eating disorders: a Delphi study

**DOI:** 10.1186/s12889-024-19989-3

**Published:** 2024-09-12

**Authors:** Diana Lemmer, Gwendolyn Mayer, Pauline Schrader, Ina Michelsen, Hans-Christoph Friederich, Stephanie Bauer

**Affiliations:** 1 Center for Psychotherapy Research, Heidelberg University Hospital, Heidelberg, Germany; 2 Ruprecht-Karls University, Heidelberg, Germany; 3 Department of General Internal Medicine and Psychosomatics, Heidelberg University Hospital, Heidelberg, Germany; 4 German Center for Mental Health (DZPG), Partner site Mannheim/Heidelberg/Ulm, Heidelberg, Germany

**Keywords:** Eating disorders, Digital mental health, Delphi study, Mental health practitioners, Expert consensus

## Abstract

**Background:**

Eating disorders (EDs) constitute a considerable burden for individuals and society, but adequate and timely professional treatment is rare. Evidence-based Digital Mental Health Interventions (DMHIs) have the potential both to reduce this treatment gap and to increase treatment effectiveness. However, their integration into routine care is lacking. Understanding practitioners’ attitudes towards DMHIs for EDs is crucial for their effective use.

**Aims:**

To investigate the consensus among German ED treatment experts on the relevance of different influencing factors for DMHI use in EDs.

**Methods:**

This Delphi study consisted of two rounds and was conducted online with an initial sample of *N* = 24 ED experts (*M*_age_=41.96, *SD*_age_=9.92, *n* = 22 female). Prior to the Delphi rounds, semi-structured qualitative telephone interviews were performed to explore participants’ attitudes, experiences, and expectations towards DMHIs. In order to construct the Delphi survey, content analysis was applied to a subset of ten interviews. A total of 63 influencing factors were identified and grouped into three main categories: contextual conditions, design, and content of DMHIs. In both Delphi rounds, the interview participants were subsequently invited to rate each of the factors with regard to their importance on 10-point scales. Group percentages and individual ratings of the first round (*n* = 23) were presented in the second round (*n* = 21). Consensus was calculated for each item (defined as IQR ≤ 2).

**Results:**

Importance ratings were high across items (*M* = 7.88, *SD* = 2.07, *Mdn* = 8). In the first round, 48% of the items reached consensus, with its most important (*Mdn* = 10) factors referring to data security, evidence base, technical requirements, usability, and specific DMHI content (psychoeducation, crisis intervention). In the second Delphi round, a consensus was reached on 73% of the items. No consensus was reached on 17 items.

**Conclusions:**

The findings on practitioners’ attitudes and priorities have relevant implications for subsequent DMHI development, dissemination, and implementation strategies, indicating that the highest-rated factors should be highlighted in the process.

**Supplementary Information:**

The online version contains supplementary material available at 10.1186/s12889-024-19989-3.

## Background

Eating disorders (EDs) are serious mental health conditions with harmful consequences for affected individuals, severely impacting their quality of life, their families, and societies at large [1]. Internationally, the main ED diagnoses of anorexia nervosa, bulimia nervosa, and binge eating disorder are estimated to affect 8.4% of women and 2.2% of men during their lifetime [2]. Their substantial disease burden and the high mortality of anorexia nervosa in particular [1, 3] underscore the importance of timely and effective treatment as a crucial public health objective. However, previous research has indicated that less than a quarter of affected individuals receive treatment [4], and the treatment gap is particularly apparent in youth [5, 6], when the majority of EDs first manifest [7]. If treatment uptake occurs, it is typically preceded by several years of ED symptomatology [8, 9]. Different structural (e.g. treatment cost, lack of access to specialists) and attitudinal barriers to help-seeking (e.g. stigma, poor mental health literacy) have been identified in previous studies, with greater impacts of attitudinal factors [10, 11].

Digital Mental Health Interventions (DMHIs), comprising a variety of technologies ranging from web-based programs and mobile applications to virtual reality and biometric trackers, are assumed to reduce some of these barriers and, in turn, to improve treatment uptake, continuity, efficacy, flexibility, and reach. For instance, a study investigating attitudes of target-users of DMHIs for EDs [12] showed that the majority of participants (> 75%) valued the advantages of not having to travel to utilize these interventions, their cost-effectiveness, and their consistent availability. Additionally, more than half of the participants reported less shame or embarrassment tied to DMHIs, indicating their potential in reducing barriers concerning the accessibility, cost, and stigma associated with face-to-face support.

Therefore, many countries have begun to develop policy frameworks regulating the integration of DMHIs into their healthcare systems in recent years [13]. Within this context, the Digital Healthcare Act was established in Germany in 2019, defining a centralized process for the evaluation of specific DMHIs (so-called “DiGAs”) and – in the case of approval – their reimbursement when they are prescribed to patients in the statutory health insurance system [14]. Within this framework, two web- and mobile-based DiGAs for bulimia nervosa and binge eating disorder have recently been made available for prescription [15, 16].

Even though the utilization of DMHIs increased during the COVID-19 pandemic [17, 18], their integration into routine care has thus far been low [19, 20]. Scepticism, concerns, and knowledge gaps in practitioners have been associated with the underutilization of DMHIs [21, 22]. While more recent research suggests that German healthcare providers generally view DMHIs for EDs positively and acknowledge their potential [23, 24], almost 90% do not feel well informed, and most have little experience with DMHIs [20]. However, clinicians are regarded as central gatekeepers to patients’ access to DMHIs [25]. Identifying and addressing practitioners’ needs and priorities is therefore essential for the successful implementation of evidence-based DMHIs.

In order to gain insight into potential approaches for improving ED-DMHI implementation, this study investigates the relevance of potential barriers and facilitators to the use of DMHIs for EDs in German ED experts with the Delphi approach. The Delphi technique involves the assessment of opinions on a particular subject in a group of experts, summarizing and presenting the initial results to them with the opportunity to re-evaluate the topics in question, and repeating this process for several rounds [26, 27]. In addition to allowing for the investigation of expert consensus, advantages of the Delphi approach lie in its efficiency as well as the maintained anonymity between participants, which are not guaranteed in alternative approaches such as focus groups. Furthermore, its particular suitability for understudied and novel research questions highlights its adequacy for the exploration of expert opinions in the field of DMHIs for EDs [26]. In particular, we aim to identify factors on which ED experts reach consensus and to provide descriptive information for each of the factors included in this study, which allows for the identification of consensual priorities among ED experts.

## Methods

### Recruitment and sample

A total of 24 clinical experts for ED treatment in Germany (i.e. clinical professionals with ED treatment experience, including staff from specialized services and specialized practitioner associations) who were contacted via e-mail participated in the present study. They were invited to participate in a semi-structured telephone interview and a subsequent Delphi study with two rounds about their perspectives on DMHIs, specifically for EDs. Potential participants were mainly identified via convenience sampling (i.e. contacts of the authors, including leading staff in specialized ED treatment services) and snowball sampling (i.e. other clinical staff members referred to the authors by the initially contacted individuals). Additional ED practitioners were identified via purposive sampling through an internet search, during which invitation e-mails were sent to specialized ED clinics and outpatient practices offering ED treatment. Furthermore, one person who fulfilled the inclusion criteria contacted the study team directly to participate. In total, 49 individuals or institutes received an invitation e-mail with a personalized code (see step 1), of which 20 did not respond (i.e. they neither consented nor refused to participate), two explicitly declined their participation (*n* = 1 without stating a reason, *n* = 1 due to a different field of expertise, namely the treatment of obesity as opposed to EDs), and three consented to participate, but were not available for the interview. We recruited ED practitioners from various primary work settings (inpatient and outpatient clinics, practices), with different therapeutic orientations (psychodynamic, cognitive behavioural, systemic), professional backgrounds (mainly medicine and psychology), and patient groups (children, adolescents, adults). In line with previous recommendations for qualitative research and average sample sizes of Delphi studies in the literature [26, 28, 29], we initially planned to recruit approximately 20 participants. As we were expecting some attrition throughout the Delphi process, we included four additional ED experts who were interested in participating. ED experts who completed the telephone interviews and both Delphi rounds were compensated with a 100 € gift card. As this study focused on the perspectives of practitioners, it did not include any patient or public involvement.

All 24 ED experts completed the telephone interviews and began participating in the first Delphi round. Of these, 23 experts (95.83%) completed the first Delphi round and 21 experts (87.50%) completed the second round. In the final sample (*N* = 21), the participants’ ages ranged between 26 and 58 years (*M* = 41.19, *SD* = 10.13), and 19 participants were female (90.48%). Table 1 illustrates further sociodemographic information of the final sample.

**Table 1:** *Sociodemographic characteristics (final Delphi round)*

Characteristic	Category	Frequency (Percentage) *n* (%)
Gender		
	Male	2 (9.52)
	Female	19 (90.48)
Type of institution		
	Hospital	16 (76.19)
	Private practice	3 (14.29)
	Hospital + private practice	1 (4.76)
	Hospital + outpatient service	1 (4.76)
Professional background		
	Psychology	12 (57.14)
	Medicine	6 (28.57)
	Pedagogics	2 (9.52)
	Social work	1 (4.76)
Professional status		
	With approbation	19 (90.48)
	In professional training	2 (9.52)
Therapeutic orientation (multiple categories possible)		
	Cognitive behavioural therapy	16 (76.19)
	Psychodynamic therapy	4 (19.05)
	Systemic therapy	2 (9.52)
Patient group		
	Adults	11 (52.38)
	Children and adolescents	8 (38.10)
	Both	2 (9.52)
**Total**		**21 (100)**

### Procedure

The present Delphi study was conducted between April and November 2022 and consisted of three steps, which are outlined in the following sections. Steps 2 and 3 were based on the methodology used by Murphy, Thorpe, Trefusis, and Kousoulis [30]. The decision to include three steps was made a priori in accordance with their methodology. Informed consent was given electronically during step 1 and was confirmed verbally on the telephone prior to the interviews.

### Step 1: telephone interviews

First, semi-structured telephone interviews were conducted with ED experts to gain insight into their experiences with and attitudes towards digital health services, DMHIs for EDs in particular. Each participant received a personal code in their invitation e-mail which served as an identifier for pseudonymous participation throughout the three steps. A study website was established. There, participants had the opportunity to view detailed information about the aims and scope of the study, to consent to participation, and to leave the researchers information about their availability and contact details for the telephone interviews. The interviews took between 35 and 60 min and followed a semi-structured guide. The interview guide comprised 14 main questions assessing: 1. sociodemographic information; 2.-3. personal use and perceived competence with respect to digital applications; 4. descriptions of the participants’ ED clienteles; 5.-6. professional experience with DMHIs; 7. perceived benefits and disadvantages of DMHIs for EDs; 8. and 11. perceived barriers and facilitators to the utilization of DMHIs in ED treatment; 9.-10. appropriate settings and treatment phases for the use of DMHIs in EDs; 12. a thought experiment about an ideal DMHI for EDs; 13. sources of information on ED treatment that participants were typically consulting; and 14. final remarks. Furthermore, each main question except for the last one (14.) contained several optional follow-up questions for the assessment of more detailed information if the respective topics were not independently mentioned during the interview. Examples include: “Which technical requirements would be necessary [to ensure an adequate integration of DMHIs in ED treatment]?” (follow-up question to main question 11) and “which design aspects would be important to you [with regard to an ideal DMHI]?”, (follow-up question to main question 12). A broad definition of DMHIs was provided at the beginning of the interviews. It included online programs and online counselling, mobile apps, augmented and virtual reality applications, biofeedback, activity trackers, and video games/ serious games. Video-based psychotherapy was also discussed in the telephone interviews. However, it was not the main focus of this study. The interviews were audio-recorded, transcribed verbatim, and deductive-inductively analysed via content analysis [31]. While the majority of codes were generated inductively from the material, some questions in the interview guide explicitly asked about specific aspects (e.g. “advantages” and “risks”), which were coded deductively in line with the guide. Two trained researchers with backgrounds in psychology (GM and DL) coded the interviews independently in an iterative process. After GM coded ten interviews while DL coded nine of the same interviews, coding was temporarily stopped in order to collaboratively create Delphi items by comparing and consensually adjusting the code system for the subsequent two steps. The decision to create the Delphi items after one-third to half of the interviews were analysed was made a priori. With regard to code saturation, this is in line with previous research [29]. Both authors agreed on a selection of preliminary codes related to desired functions and properties of DMHIs for EDs (including design and functionality aspects) as well as general conditions and requirements necessary for their utilization to be used in the Delphi study. These categories were chosen because of their focus on practical suggestions and ideas relevant to the implementation of DMHIs rather than general attitudes (e.g. perceived advantages and disadvantages of DMHIs). Along with the complete interview guidelines, the final and complete results of all 24 interviews with 9 major codes and subcodes on two levels are reported elsewhere [32].

### Step 2: first Delphi round – online survey

Thematic codes from the interviews were used to generate items about influencing factors for the implementation of DMHIs in ED treatment. A total of 63 items were extracted and the categories “content” (for those affected by EDs: 16 items, for informal caregivers such as family members: 4 items, for practitioners: 5 items), “design” (10 items), and “contextual conditions” (28 items) emerged. The category “content” comprised topics and functions that DMHIs might include, such as crisis intervention elements (e.g. safety plan, emergency contact list; item 34), psychoeducation for informal caregivers (item 47), and the ability to provide feedback to affected individuals as practitioners (item 51). Items related to the “design” category addressed elements concerning the layout, composition, and style of DMHIs that might have an impact on user experiences, including aesthetics (item 55), the involvement of affected individuals in the creation of DMHIs (item 59), and usability (item 61). “Contextual conditions” referred to external requirements potentially impacting the suitability of DMHI utilization, such as a stable internet connection (item 3), cost coverage by insurance providers (item 14), or data protection and data security (item 15). Additionally, two optional open text fields were included to allow for comments and other potential factors that were not included in this study. A website was established for participants to rate each of the 63 aspects with regard to their respective importance for the implementation of ED-DMHIs on a scale from 1="completely unimportant” to 10="utterly important”. The software ASMO was used to implement the online-survey [33]. Its duration amounted to approximately 5 min. The whole sample (*N* = 24) responded to this online survey. However, one of the participants did not complete it, and one other ED expert participated while the authors were already analysing the data for the second Delphi round, i.e. preparing the frequency distributions of the whole sample’s ratings. We were thus able to use the complete datasets of 22 participants and the incomplete data of one participant to calculate the frequency distributions for the next step.

### Step 3: second Delphi round – reviewing responses and iterative rating

**Fig. 1:**
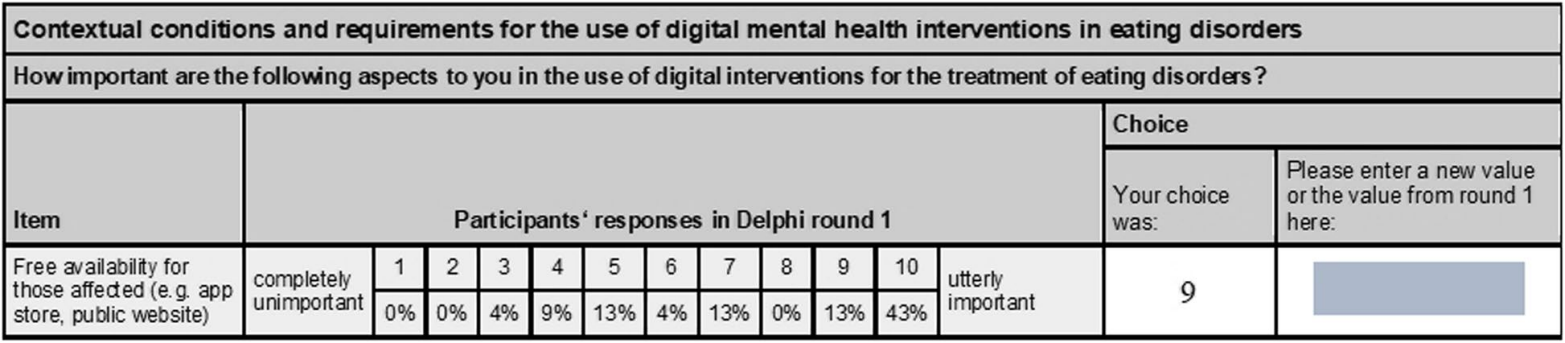
Example of step 3-assessment Note. In step 2, participants received the same instructions to rate each item based on its perceived importance as in step 3. Response percentages were only presented in step 3

The whole sample of 24 ED experts was invited to participate in the second Delphi round. For each participant, an individual editable pdf file was created and sent via e-mail. It contained the same 63 items from the first Delphi round and one optional open text field for suggestions and feedback. For each item, the group-percentage scores as well as the participants’ individual ratings were presented. ED experts were asked to rate each of the items again and to send their responses back via e-mail (see Fig. 1). In total, 21 participants completed this step.

### Data analysis

For each of the 63 items, descriptive statistics (percentages, means, standard deviations, ranges, interquartile ranges) were calculated. Consensus was defined as an interquartile range (IQR) of ≤ 2 on the 10-point scale [30]. Statistical analyses were performed with R 4.3 [34].

## Results

Across items, the average importance ratings were high in both Delphi rounds (steps 2 and 3; first round: *M* = 7.71, *SD* = 2.17, *Mdn* = 8, range: 1–10; second round: *M* = 7.88, *SD* = 2.07, *Mdn* = 8, range: 1–10). After the first round, a consensus was reached for 30 items (47.62%). Two items (3.17%) were consensually rated with a median importance score of 10. Detailed results of the first Delphi round can be found in Additional File 1. After the second round, consensus was reached for 46 items (73.02%), of which 7 items (11.11%) were rated with a median score of 10. Table 2 shows the descriptive statistics for each of the 63 items of the second Delphi round.

**Table 2:** Items and descriptive statistics of the final Delphi round (step 3)

Item	M	SD	Mdn	min	max	IQR
Contextual conditions						
1. Free availability for those affected (e.g. app store, public website) *(access)*	8.10	2.26	9	4	10	3
2. Information about data storage ^a^*(data security)*	8.95	1.20	9	7	10	2
3. **Stable internet connection**^**a, b**^***(technology)***	**9.24**	**1.14**	**10**	**7**	**10**	**1**
4. Indicated use (e.g. diagnosis, functioning) ^a^*(evidence/ indication)*	8.10	1.51	8	5	10	2
5. Technical contact person for practitioners (e.g. for training, maintenance of equipment and software) ^a^*(staff)*	8.62	1.69	9	5	10	2
6. Willingness of the team (e.g. in clinics) to implement the interventions ^a^*(staff)*	8.52	1.03	9	5	10	1
7. **No substitute for conventional professional treatment**^**a, b**^***(evidence/ indication)***	**9.19**	**1.21**	**10**	**7**	**10**	**1**
8. Reasonable extent of use (enough time for practice, but no excessive use) ^a^*(setting)*	7.57	1.63	8	4	10	2
9. Long-term access to content for those affected ^a^*(technology)*	7.10	2.07	8	2	10	1
10. Training and education opportunities for practitioners ^a^*(staff)*	8.29	1.27	8	5	10	1
11. No data storage *(data security)*	7.90	2.41	9	3	10	4
12. Free of charge for those affected ^a^*(finances)*	8.14	1.93	8	4	10	2
13. Compatibility with different devices (e.g. smartphone, tablet, computer) ^a^*(technology)*	8.43	2.27	9	2	10	2
14. Cost coverage by health insurance *(finances)*	7.48	2.18	8	1	10	3
15. **Ensuring data protection and data security**^**a, b**^***(data security)***	**9.29**	**1.49**	**10**	**5**	**10**	**0**
16. Compensation of additional efforts for practitioners ^a^*(finances)*	7.62	1.88	8	2	10	2
17. Easy access for practitioners (e.g. low barriers in acquisition) ^a^*(access)*	8.76	1.14	9	6	10	2
18. Prescription requirement ^a^*(access)*	2.95	1.43	3	1	6	1
19. Sufficient evidence for effectiveness *(evidence/ indication)*^a^	7.81	1.81	8	2	10	2
20. Availability of technical equipment in therapeutic setting (e.g. work phone, tablets) ^a^*(technology)*	8.43	1.96	9	1	10	1
21. Use during treatment transitions (e.g. from inpatient to outpatient setting) ^a^*(setting)*	8.52	1.03	9	6	10	1
22. Independent use (self-help) ^a^*(setting)*	7.24	1.64	7	3	10	2
23. Use in prevention *(setting)*	6.57	2.27	7	2	10	3
24. Use for bridging waiting periods ^a^*(setting)*	8.29	0.96	8	7	10	1
25. Blended treatment, outpatient care ^a^*(setting)*	7.90	1.67	8	4	10	1
26. Blended treatment, inpatient care ^a^*(setting)*	6.90	2.32	8	1	10	2
27. Safe and calm environment for affected individuals, no disruptions *(setting)*	6.95	2.36	7	1	10	3
28. Self-experience/ testing opportunities for practitioners *(staff)*	7.14	2.10	7	3	10	4
**Functions and content (individuals affected by EDs)**						
29. Mindfulness and relaxation	6.38	2.13	6	4	10	4
30. Structuring daily routine (e.g. meal plans) ^a^	8.76	1.37	9	6	10	2
31. Diagnostics, screening	5.90	1.87	6	3	9	4
32. Reminders (e.g. for protocols, meal times) ^a^	8.29	1.27	8	5	10	1
33. Exposition, confrontation (e.g. meal situations, body image) ^a^	8.10	1.64	8	2	10	1
34. **Crisis intervention (e.g. safety plan**,** emergency contact list)**^**a, b**^	**8.62**	**1.96**	**10**	**3**	**10**	**2**
35. Motivation, affirmation (e.g. positive feedback for completed tasks) ^a^	8.81	0.81	9	8	10	1
36. **Personalized feedback (e.g. individual screening results)**^**a, b**^	**9.19**	**1.29**	**10**	**5**	**10**	**2**
37. **Psychoeducation**^**a, b**^	**8.90**	**1.55**	**10**	**5**	**10**	**2**
38. Activating resources (e.g. strengthening social skills) ^a^	8.86	1.11	9	7	10	2
39. Skills training (e.g. emotion regulation) ^a^	8.90	1.22	9	7	10	2
40. Reflection (e.g. diary) ^a^	8.00	1.67	8	4	10	1
41. Exercises, homework ^a^	8.10	1.09	8	6	10	1
42. Protocols (e.g. meals, weight, movement) ^a^	8.24	2.07	8	1	10	2
43. Measuring symptom progression (e.g. mood, weight/ shape concerns) ^a^	8.48	1.29	8	5	10	2
44. Suggestions for movement and exercise	6.33	2.61	6	2	10	3
**Functions and content (informal caregivers)**						
45. Recommended action (e.g. meal plans, decision aids)	7.14	2.33	7	2	10	4
46. Interactive area (e.g. contributing to family plans, adding components to the affected individual’s disorder model)	5.90	2.74	6	1	10	4
47. Psychoeducation	7.81	2.40	9	2	10	4
48. Access to selected components of the intervention	6.48	2.56	7	2	10	5
**Functions and content (practitioners)**						
49. Individual activation of content by practitioners	8.19	1.63	8	5	10	3
50. Access to entries of affected individuals (e.g. weight logs, completed exercises) ^a^	8.48	1.86	9	2	10	2
51. Ability to provide feedback to affected individuals ^a^	8.67	1.53	9	6	10	2
52. Interactive area (e.g. digital whiteboard with shared access for both practitioners and affected individuals) ^a^	8.33	1.62	8	4	10	2
53. Videoconferencing with affected individuals	7.48	2.60	8	2	10	4
**Design**						
54. Age-appropriate design ^a^	8.67	0.97	9	7	10	1
55. Attractive design, aesthetics (modern, pleasant colors) ^a^	8.81	1.03	9	7	10	2
56. Personal guidance (practitioners, counsellors) ^a^	8.48	1.54	8	4	10	2
57. Guidance through interactive chatbot ^a^	6.19	1.78	6	2	10	2
58. Gamification, fun factor (e.g. playfully learning to estimate meal portions) ^a^	6.81	2.06	7	1	10	1
59. Co-creation with affected individuals	7.57	2.48	8	3	10	3
60. Interactivity (e.g. visual illustration of weight changes, whiteboard) ^a^	8.00	1.73	8	4	10	2
61. **Usability (easy and intuitive to use**,** clear)**^**a, b**^	**9.71**	**0.56**	**10**	**8**	**10**	**0**
62. Personalized design (e.g. avatars, personalized content) ^a^	6.19	2.29	6	1	10	2
63. Private area (access only for affected individuals)	7.43	2.23	8	3	10	3

Note. Parenthesized and italicized terms represent subcategories of the main category “contextual conditions”

^a^ fulfils consensus criterion (IQR ≤ 2)

^b^ items with highest importance ratings (*Mdn* = 10). These items are additionally bolded

Throughout the two Delphi rounds, two participants provided comments in the open text fields. One participant stated in round 1:

I find it difficult to answer the questions generally; I would actually provide different answers depending on the type of applications, the patient group, and the context.

(Medical specialist working with adult outpatients in a clinic, psychodynamic approach, 58, female)

In round 2, another participant provided the following response:

Nothing new, but I view the area of ‘functions and content for informal caregivers’ critically in that it involves a lot of interpersonal dynamics. Considering the complexity and the fact that eating disorders always revolve a lot around taking personal responsibility and developing autonomy, I do not find it reasonable to involve relatives digitally. It is precisely the autonomous utilization by those affected and the highly individual process they could better engage in as a result that I am expecting a large effect from.

(Child and adolescent psychotherapist working with outpatients at a practice, CBT and systemic approaches, 45, female)

## Discussion

### Principal findings

This Delphi study assessed the perspectives of German clinical ED experts on the importance of different factors regarding contextual conditions, content, and design for the implementation of DMHIs in ED treatment.

### Consensus

After the second Delphi round, consensus (IQR ≤ 2) was reached for the majority of items (73%). We identified seven key aspects that were consensually rated as the most important (*Mdn* = 10). A stable internet connection, data security and data protection, as well as refraining from the use of DMHIs as a substitute for conventional professional treatment were consensually rated as the most important contextual facilitators for the implementation of DMHIs. These findings are in line with previous research, which identified technical issues as a critical barrier to the use of DMHIs [35, 36]. Furthermore, previous findings underscore the need for data security [23, 37] and a preference for blended treatment as compared to stand-alone applications in both practitioners and other stakeholders, such as potential users [22, 24, 38]. On the flipside, some evidence suggests that data privacy and security receive limited attention from digital health intervention users [36], which could be an indicator of differing priorities in different groups of stakeholders. Moreover, usability was consensually rated as the most important design aspect, which fits into the current frame of research since effort expectancy (i.e. the expected ease of use) significantly predicts behavioural intentions to utilize digital health interventions [39] and ease of use has been identified as a key contributor to a positive user experience [36]. With regard to content and functionalities for affected individuals, psychoeducation, crisis intervention, and personalization were rated as the most important aspects. Since the delivery of psychoeducational and emergency information can be easily implemented in a digital format and practitioners were more likely to refer patients to web-based psychoeducational interventions than more complex DMHIs in previous studies [37], these findings are consistent with prior research. Personalization further contributes to a positive user experience [36] and the need for tailored ED-DMHIs has been expressed previously [23].

On the other hand, prescription requirement was consensually rated as the least important contextual condition. While certified digital health applications (“DiGAs”) listed in the German DiGA directory have been found to receive more positive app store ratings and reviews than unregulated mobile health apps do [36], the requirement for prescription in itself did not appear to be a priority in this sample.

### No consensus

No consensus (IQR > 2) was reached on specific contextual conditions, such as the opportunity to self-experience and pre-test DMHIs among practitioners, free accessibility for affected individuals (e.g. via app stores or public websites), and the use in a prevention setting. No consensus was reached on any of the functions or contents for informal caregivers, specific functions for practitioners (e.g. videoconferencing), and for those affected (e.g. suggestions for movement/exercise). Furthermore, the design elements co-creation (i.e. participatory design that involves affected individuals) and a private section for affected individuals (i.e. no other potential user-group such as practitioners would be able to access these functions or data inputs) did not elicit consensus. Taken together, the importance of these factors might depend on the specific needs and aims of individual treatment plans, which might make general statements challenging. For instance, as one of the participants stated during step 3, contents and functions for informal caregivers can be viewed critically as they contradict the development of autonomy during treatment. On the other hand, interpersonal and potentially harmful influences in, for instance, family settings, were addressed in the interviews (step 1) and a need for psychoeducational interventions for informal caregivers was mentioned in order to facilitate informal support during treatment. Initial studies on DMHIs for informal caregivers point towards beneficial effects for parents (e.g. stress release, increased confidence in parenting abilities) [40, 41] and the ED symptoms of their children [42, 43]. The use of DMHIs and specific elements should therefore be tailored to individual needs.

### Limitations

One limitation of this study lies in the broad definition of DMHIs, which included a range of technologies (e.g. online programs, smartphone apps, virtual reality applications). On the one hand, this allowed for the investigation of more general, common factors across different DMHI types. On the other hand, this was accomplished at the expense of potentially missing DMHI-specific factors.

Related to this, the Delphi items referred to all EDs. While little is known about the perceptions of DMHIs for EDs among practitioners, which underlines the value of identifying core factors across EDs, future research should investigate differences and specificities between different types of EDs. For instance, item 44 “suggestions for movement and exercise” reflects one potential function for affected individuals that could be useful in some contexts and potentially harmful in others, depending on the individual needs of those affected by an ED.

Furthermore, practitioners who were female, CBT-trained, and who provided inpatient treatment were overrepresented in this sample as compared to other groups of practitioners, which limits the generalizability of results. Moreover, the interviews indicated a general interest and openness towards DMHIs in our sample, which is consistent with previous findings [23, 24]. However, it is plausible that practitioners with more positive views towards DMHIs were more inclined to participate, and sceptical voices might be underrepresented in our study, which was potentially amplified by our recruitment procedures which focused on convenience and snowball sampling. Future research should thus strive to include a wider range of perspectives.

In this regard, other studies point towards a need for targeted information materials on DMHIs for different groups of healthcare providers (e.g. general practitioners, specialists) [20], other relevant stakeholders (e.g. patients, policy makers), and different healthcare systems [21, 22]. Identifying specificities and similarities between these groups and settings with regard to their priorities allows for the development of targeted strategies to successfully implement evidence-based DMHIs in routine care. While the present study exclusively aimed to address the perspectives of practitioners with an expertise in the field of EDs, other stakeholders, particularly patients and informal caregivers, are also essential for the implementation of DMHIs. Future studies should therefore focus on their perspectives.

## Conclusions

This Delphi study identified the relevance of different influencing factors for the implementation of DMHIs for EDs in a sample of German ED experts. To improve the implementation of DMHIs in routine care, the study results suggest that usability, data security, psychoeducational and crisis intervention content, which were the highest-rated factors on which consensus was reached, should be highlighted in the development and dissemination of DMHIs. More research is needed to identify preferences and priorities for targeted interventions (i.e. specific technologies, EDs), to involve other relevant perspectives (e.g. patients), and to assess similarities and differences between different health care systems and cultures.

## Supplementary Information

Supplementary Material 1.

## Data Availability

Individual anonymous participant data underlying the results of this publication (text, tables, figures, appendices) and statistical code will be shared during the period of 3 months to 5 years following article publication with researchers who provide a methodologically sound proposal to the corresponding author.

## Abbreviations

CBT: Cognitive behavioural therapy
DiGA: Digitale Gesundheitsanwendung (digital health application)
DMHI: Digital mental health intervention
ED: Eating disorder
