# Supplementary material for: Experts’ views on the implementation of digital interventions for eating disorders: a Delphi study
Source: BMC Public Health. 2024 Sep 12;24:2486. doi: 10.1186/s12889-024-19989-3 (PMC11396553; doi:10.1186/s12889-024-19989-3)
Supplement: Supplementary file 1 — Supplementary Material 1. [file 12889_2024_19989_MOESM1_ESM.docx]

| **Additional file 1. Items and descriptive statistics of the first Delphi round (step 2).** | | | | | | | |
| --- | --- | --- | --- | --- | --- | --- | --- |
| **item** | ***n*** | ***M*** | ***SD*** | ***Mdn*** | ***min*** | ***max*** | ***IQR*** |
| **Contextual conditions** | | | | | | | |
| 1. Free availability for those affected (e.g. app store, public website) *(access)* | 24 | 7.63 | 2.60 | 9 | 3 | 10 | 5 |
| 1. Information about data storage *(data security)* | 24 | 8.58 | 1.61 | 9 | 5 | 10 | 3 |
| 1. Stable internet connection ^a^ *(technology)* | 24 | 8.79 | 1.35 | 9 | 6 | 10 | 2 |
| 1. Indicated use (e.g. diagnosis, functioning) *(evidence/ indication)* | 24 | 8.21 | 1.53 | 8 | 5 | 10 | 2.25 |
| 1. Technical contact person for practitioners (e.g. for training, maintenance of equipment and software) *(staff)* | 24 | 8.42 | 1.61 | 9 | 5 | 10 | 3 |
| 1. Willingness of the team (e.g. in clinics) to implement the interventions ^a^ *(staff)* | 24 | 8.42 | 1.77 | 9 | 1 | 10 | 1 |
| 1. No substitute for conventional professional treatment *(evidence/ indication)* | 24 | 8.08 | 2.30 | 9 | 1 | 10 | 3 |
| 1. Reasonable extent of use (enough time for practice, but no excessive use) ^a^ *(setting)* | 24 | 7.50 | 1.79 | 8 | 4 | 10 | 2 |
| 1. Long-term access to content for those affected *(technology)* | 24 | 6.71 | 2.35 | 8 | 2 | 9 | 2.25 |
| 1. Training and education opportunities for practitioners ^a^ *(staff)* | 24 | 7.79 | 1.86 | 8 | 3 | 10 | 2 |
| 1. No data storage *(data security)* | 24 | 7.33 | 2.55 | 7.5 | 3 | 10 | 5 |
| 1. Free of charge for those affected *(finances)* | 24 | 7.83 | 1.95 | 8 | 4 | 10 | 4 |
| 1. Compatibility with different devices (e.g. smartphone, tablet, computer) *(technology)* | 24 | 8.00 | 2.38 | 9 | 2 | 10 | 3.25 |
| 1. Cost coverage by health insurance *(finances)* | 24 | 7.63 | 2.20 | 8 | 1 | 10 | 2.25 |
| 1. **Ensuring data protection and data security ^a,b^ *(data security)*** | 24 | 9.17 | 1.43 | 10 | 5 | 10 | 1.25 |
| 1. Compensation of additional efforts for practitioners ^a^ *(finances)* | 24 | 7.54 | 2.08 | 8 | 2 | 10 | 2 |
| 1. Easy access for practitioners (e.g. low barriers in acquisition) ^a^ *(access)* | 24 | 8.46 | 1.38 | 9 | 5 | 10 | 1.25 |
| 1. Prescription requirement *(access)* | 24 | 3.75 | 2.45 | 3 | 1 | 10 | 2.25 |
| 1. Sufficient evidence for effectiveness *(evidence/ indication)* | 24 | 7.54 | 1.91 | 8 | 2 | 10 | 3 |
| 1. Availability of technical equipment in therapeutic setting (e.g. work phone, tablets) ^a^ *(technology)* | 24 | 8.29 | 2.14 | 9 | 1 | 10 | 1.25 |
| 1. Use during treatment transitions (e.g. from inpatient to outpatient setting) ^a^ *(setting)* | 24 | 8.04 | 1.83 | 9 | 1 | 10 | 2 |
| 1. Independent use (self-help) ^a^ *(setting)* | 24 | 6.75 | 1.89 | 7 | 3 | 10 | 2 |
| 1. Use in prevention *(setting)* | 24 | 6.67 | 2.53 | 7 | 1 | 10 | 4 |
| 1. Use for bridging waiting periods ^a^ *(setting)* | 24 | 7.71 | 2.12 | 8 | 1 | 10 | 2 |
| 1. Blended treatment, outpatient care ^a^ *(setting)* | 24 | 7.42 | 2.26 | 8 | 1 | 10 | 2 |
| 1. Blended treatment, inpatient care *(setting)* | 24 | 6.54 | 2.64 | 7.5 | 1 | 10 | 3.25 |
| 1. Safe and calm environment for affected individuals, no disruptions *(setting)* | 24 | 7.04 | 2.37 | 7.5 | 1 | 10 | 3.25 |
| 1. Self-experience/ testing opportunities for practitioners *(staff)* | 24 | 6.96 | 2.40 | 7 | 1 | 10 | 4 |
| **Functions and content (individuals affected by EDs)** | | | | | | | |
| 1. Mindfulness and relaxation | 23 | 6.48 | 2.41 | 6 | 2 | 10 | 4 |
| 1. Structuring daily routine (e.g. meal plans) ^a^ | 23 | 8.39 | 1.64 | 8 | 4 | 10 | 2 |
| 1. Diagnostics, screening | 23 | 6.09 | 2.27 | 6 | 1 | 10 | 3.5 |
| 1. Reminders (e.g. for protocols, meal times) ^a^ | 23 | 8.13 | 1.71 | 8 | 3 | 10 | 1 |
| 1. Exposition, confrontation (e.g. meal situations, body image) ^a^ | 23 | 7.87 | 1.74 | 8 | 2 | 10 | 1 |
| 1. Crisis intervention (e.g. safety plan, emergency contact list) ^a^ | 23 | 8.39 | 1.95 | 9 | 3 | 10 | 2 |
| 1. Motivation, affirmation (e.g. positive feedback for completed tasks) ^a^ | 23 | 8.78 | 0.85 | 9 | 7 | 10 | 1 |
| 1. Personalized feedback (e.g. individual screening results) ^a^ | 23 | 8.91 | 1.44 | 9 | 4 | 10 | 2 |
| 1. Psychoeducation | 23 | 8.65 | 1.58 | 9 | 5 | 10 | 2.5 |
| 1. Activating resources (e.g. strengthening social skills) ^a^ | 23 | 8.74 | 1.18 | 9 | 7 | 10 | 2 |
| 1. Skills training (e.g. emotion regulation) ^a^ | 23 | 8.61 | 1.31 | 9 | 5 | 10 | 2 |
| 1. Reflection (e.g. diary) ^a^ | 23 | 7.96 | 1.52 | 8 | 4 | 10 | 2 |
| 1. Exercises, homework ^a^ | 23 | 7.78 | 1.70 | 8 | 3 | 10 | 2 |
| 1. Protocols (e.g. meals, weight, movement) | 23 | 8.35 | 1.43 | 8 | 6 | 10 | 2.5 |
| 1. Measuring symptom progression (e.g. mood, weight/ shape concerns) ^a^ | 23 | 8.26 | 1.36 | 8 | 5 | 10 | 1.5 |
| 1. Suggestions for movement and exercise | 23 | 6.61 | 2.57 | 6 | 2 | 10 | 4 |
| **Functions and content (informal caregivers)** | | | | | | | |
| 1. Recommended action (e.g. meal plans, decision aids) | 23 | 7.52 | 2.23 | 8 | 2 | 10 | 3.5 |
| 1. Interactive area (e.g. contributing to family plans, adding components to the affected individual’s disorder model) | 23 | 6.70 | 2.69 | 7 | 1 | 10 | 4 |
| 1. Psychoeducation | 23 | 7.91 | 2.61 | 9 | 2 | 10 | 3 |
| 1. Access to selected components of the intervention | 23 | 6.61 | 2.61 | 7 | 1 | 10 | 5 |
| **Functions and content (practitioners)** | | | | | | | |
| 1. Individual activation of content by practitioners | 23 | 7.87 | 1.79 | 8 | 4 | 10 | 3 |
| 1. Access to entries of affected individuals (e.g. weight logs, completed exercises) | 23 | 8.09 | 2.07 | 9 | 2 | 10 | 3 |
| 1. Ability to provide feedback to affected individuals ^a^ | 23 | 8.61 | 1.50 | 9 | 5 | 10 | 2 |
| 1. Interactive area (e.g. digital whiteboard with shared access for both practitioners and affected individuals) ^a^ | 23 | 7.91 | 1.83 | 8 | 4 | 10 | 2 |
| 1. Videoconferencing with affected individuals | 23 | 7.52 | 2.41 | 8 | 2 | 10 | 3.5 |
| **Design** | | | | | | | |
| 1. Age-appropriate design ^a^ | 23 | 8.57 | 1.08 | 9 | 6 | 10 | 1 |
| 1. Attractive design, aesthetics (modern, pleasant colors) ^a^ | 23 | 8.57 | 1.27 | 9 | 6 | 10 | 2 |
| 1. Personal guidance (practitioners, counsellors) ^a^ | 23 | 8.35 | 1.40 | 8 | 5 | 10 | 2 |
| 1. Guidance through interactive chatbot | 23 | 6.26 | 2.03 | 6 | 2 | 10 | 2.5 |
| 1. Gamification, fun factor (e.g. playfully learning to estimate meal portions) | 23 | 6.61 | 2.19 | 7 | 1 | 10 | 2.5 |
| 1. Co-creation with affected individuals | 23 | 7.17 | 2.57 | 8 | 2 | 10 | 4 |
| 1. Interactivity (e.g. visual illustration of weight changes, whiteboard) ^a^ | 23 | 7.87 | 1.71 | 8 | 4 | 10 | 2 |
| 1. **Usability (easy and intuitive to use, clear) ^a,b^** | 23 | 9.52 | 0.73 | 10 | 8 | 10 | 1 |
| 1. Personalized design (e.g. avatars, personalized content) | 23 | 6.22 | 2.52 | 6 | 1 | 10 | 3 |
| 1. Private area (access only for affected individuals) | 23 | 7.09 | 2.66 | 7 | 1 | 10 | 4 |

Note. Parenthesized and italicized terms represent subcategories of the main category “contextual conditions”.

^a^ fulfills consensus criterion (IQR ≤ 2).

^b^ items with highest importance ratings (*Mdn*=10). These items are additionally bolded.
